# A narrative review of research advances in gut microbiota and microecological agents in children with attention deficit hyperactivity disorder (ADHD)

**DOI:** 10.3389/fpsyt.2025.1588135

**Published:** 2025-05-23

**Authors:** Yang Liu, Panpan Zhang, Hao Sun

**Affiliations:** Department of Pediatrics, Dalian Municipal Women and Children’s Medical Center (Group), Affiliated to Dalian Medical University, Dalian, Liaoning, China

**Keywords:** ADHD, gut microbiota, microecological agents, children, gut-brain axes

## Abstract

The role of gut microecology in attention deficit hyperactivity disorder (ADHD) has garnered growing attention. Studies have suggested a potential link between ADHD development and an imbalance in gut microbiota composition. This review aims to analyze the characteristics of the gut microbiota in children with ADHD, explore how changes in the gut microbiota affect ADHD through nervous, neuroendocrine, and immune pathways, and discuss the potential application of microecological agents and fecal microbiota transplantation in the prevention and treatment of ADHD in children. Pubmed, Google Scholar, EBSCO, Scopus and Medline were utilized to conduct searches using the following key terms:Attention Deficit Hyperactivity Disorder OR ADHD AND gut microbiota OR probiotics OR prebiotics OR synbiotics OR fecal microbiota transplantation OR FMT. Studies published in English from all years were included. A thorough review of numerous papers and their references was conducted to identify relevant articles. Sorting and analysis revealed that the gut microbiota of children with ADHD has changed to some extent, and targeting the gut microbiota, using microecological agents or fecal microbiota transplantation, especially in combination with central nervous system stimulants, may provide additional benefits for children with ADHD.

## 1 Introduction

Attention deficit hyperactivity disorder (ADHD), a prevalent chronic neurodevelopmental disorder in childhood, is primarily characterized by attention deficits and/or hyperactive impulses, disproportionately related to developmental stage (1). Children with ADHD typically exhibit poor academic performance and interpersonal relationships, and the impairment usually persists into adolescence and beyond (2). Specifically, 40-60% of ADHD patients experience partial relief, while 15% still meet diagnostic criteria in adulthood (3). ADHD often co-exists with other neurodevelopmental disorders, such as tic disorders, intellectual disability, autism spectrum disorders (ASD), communication disorders, and specific learning or motor disorders (e.g. developmental coordination disorder, reading ability impairment) (4–6).

In 2012, an epidemiological study found that 6.8% of children and adolescents in Spain had ADHD (7). In 2015, a meta-analysis reported a global ADHD prevalence rate of 7.2% (8). In 2018, the global prevalence of ADHD ranged from 2% to 7%, with an average of about 5% (9). In addition, at least 5% of children have hyperactivity, inattention, and impulsivity that are less severe than the diagnostic criteria for ADHD (9). In the same year, a study by the American National Survey of Children’s Health revealed ADHD diagnoses in 8.4% of children between the ages of 2–17 (10). Another meta-analysis published in 2018 revealed a 5.6% prevalence of ADHD in Chinese children, notably more common in boys (7.7%) compared to girls (3.4%) (11). The latest meta-analysis of global ADHD prevalence in 2023 showed that 7.6% of children between 3 and 12 years and 5.6% of adolescents aged 12 to 18 years were diagnosed with ADHD (12). It can be seen that the prevalence of ADHD in children is high and rising. ADHD not only significantly impacts the quality of life for children and their families, but also imposes a heavy burden on society (13–16).

The underlying cause of ADHD is unknown, but first-degree relatives of ADHD patients have a five to ninefold higher risk of developing ADHD than those without (17). Twin studies show ADHD heritability is about 76% (18). A meta-analysis identified serotonin transporter gene, dopamine transporter gene, and norepinephrine transporter gene as significant positional candidate genes for ADHD status (19, 20). In 2023, a genome-wide association study (GWAS) of ADHD reported 27 significant loci, highlighting 76 potential risk genes that are particularly enriched during early brain development (21). At the same time, a meta-analysis of GWAS showed that approximately 22% of the genetic predisposition was caused by single nucleotide polymorphisms (SNPs) inheritance (22, 23). SNPs are the most frequently occurring genetic variation in the human genome, which are important markers in many studies that link sequence variations to phenotypic changes (24). However, attributing the onset of ADHD entirely to genetic factors cannot explain the high incidence of ADHD in recent years, and attention also needs to be paid to between other factors, such as environmental factors of ADHD.

Environmental factors have a multifaceted impact on ADHD, involving a range of prenatal, perinatal, and postnatal factors. Prenatal factors include maternal stress, substance exposure and environmental toxins. Studies have shown that children whose mothers experienced moderate to severe stress during pregnancy exhibited more severe ADHD symptoms compared to those with less stressful prenatal environments. Mothers who drink or smoke during pregnancy, or who are exposed to lead can interfere with fetal brain development and increase the likelihood of neurobehavioral disorders, and thereby increase the risk of ADHD in their offspring (25, 26). Perinatal factors such as preterm birth, low birth weight and caesarean section are associated with a higher incidence of ADHD in offspring (27). Postnatal factors like breastfeeding, exposure to pollutants and chemicals, allergies and gut microecological disorders can exacerbate ADHD symptoms (28). Shorter breastfeeding periods or lack of exclusive breastfeeding may increase the risk of ADHD symptoms in children (29). Studies have found a high prevalence of allergic comorbidity among children with ADHD (30, 31). Several studies have found that children with allergic diseases have a significantly increased risk of co-occurring ADHD, with risk increasing with the severity and number of allergy symptoms (32, 33). Imbalance of gut microbiota through the gut-brain axis has been implicated in ADHD and is an increasingly studies area (34, 35). In conclusion, environmental factors play a significant role in the development and exacerbation of ADHD symptoms in children.

Regarding ADHD symptom management, behavioral management should be the initial treatment for children <6 with ADHD, medication is recommended as the first-line treatment for ≥6 with ADHD, classified as both stimulants (methylphenidate and amphetamine formulations) and non-stimulants (atomoxetine, viloxazine, clonidine, and guanfacine) (2, 36, 37). Numerous clinical trials have demonstrated the efficacy of stimulants like methylphenidate and amphetamine in reducing ADHD symptoms in short-term treatment (38). A meta-analysis of more than 10,000 children and adolescents found moderate to large effects of methylphenidate and amphetamine based on clinician and teacher assessments of symptom changes (38). However, there are potential side effects of medication, such as insomnia, loss of appetite, nausea, and emotional instability, etc (39). Additionally, 20-35% of children with ADHD poorly respond to treatment (40), and reduced medication adherence can also result from long-term administration tolerance (41). Furthermore, concerns exist about the selection and use of stimulants medications in three areas: the impact of long-term treatment on growth, the likelihood of drug abuse and dependence, and the chance of cardiovascular incidents owing to sympathomimetic properties (2). Thus, the limitations of ADHD medication highlight the importance of continuing to search for new and improved ways to management ADHD.

In recent years, the concept of gut microbiota has taken center stage in scientific research, particularly in understanding its role in the intricate bidirectional communication between the brain and gut. This microscopic ecosystem has been discovered to potentially influence the development of ADHD via multiple distinct pathways (42–44). As understanding of these relationships deepens, the use of microecological agents, which are substances that help to regulate and balance the gut microbiota, has garnered significant attention among researchers and clinicians as a potential therapeutic approach. These agents hold promise as a novel and natural method to regulate gut microbiota imbalances often observed in ADHD patients.

This review examines the gut-brain axis, the characteristics of gut microecology in children with ADHD, the possible mechanisms of gut microecology disorders affecting ADHD and the application of microecological agents in ADHD, with the goal of providing new insights for the prevention and treatment of ADHD.

## 2 Methods

To investigate the associations between attention deficit hyperactivity disorder, gut microecology, and microecological agents in children, we conducted a comprehensive literature review, which included both clinical and experimental studies. Searchers were carried out utilizing databases like EBSCO, Pubmed, Scopus, Google Scholar, Medline, focusing on the following key terms: gut-brain axis OR (gut microbiota AND central nervous system) OR (gut microbiota AND enteric nervous system AND central nervous system); (attention deficit hyperactivity disorder OR ADHD) AND gut microbiota; (attention deficit hyperactivity disorder OR ADHD) AND (probiotics OR prebiotics OR synbiotics); (attention deficit hyperactivity disorder OR ADHD) AND (fecal microbiota transplantation OR FMT). Research spanning all years were incorporated. The search was limited to published by English.

## 3 Gut-brain axis

The human gastrointestinal tract harbors a diverse ecosystem, spanning from the oral cavity to the rectum, with a surface area of 150–200 m^2^ providing ample habitat for microorganisms. The gut content contains a high number of bacteria, ranging from 100,000 to 100 billion per milliliter. Gut microecology has a profound effect on various gastrointestinal and non-gastrointestinal functions. These microorganisms play crucial roles in digestion and absorption of various nutrients, such as proteins, carbohydrates, bile acids, vitamins, and so on (45, 46). Furthermore, gut microbiota has been reported to influence non-gastrointestinal functions, such as brain development, immunity, and maturation of the endocrine system (47–49).

The gut has been found to interact with the brain along what is known as the gut-brain axis (50). This interaction occurs via multiple pathways including neural, immune, endocrine and metabolic. Gut microbiota can produce neurotransmitters directly or indirectly, via the host’s biosynthetic routes, and imbalances in this process can lead to disorders in neurotransmitter and neurodevelopmental function (51, 52). Beneficial bacteria like *Bifidobacterium*, *Lactobacillus*, and *Bacillus* produce various neurotransmitters such as dopamine (DA), norepinephrine (NE), 5-hydroxytryptamine (5-HT), gamma-aminobutyric acid (GABA), acetylcholine (ACh), and histamine, among others (53). The aforementioned neurotransmitters enter the central nervous system (CNS) via the enteric nervous system (ENS) and influence the physiological functions of the brain (54, 55). Research indicates that *Bifidobacterium* and certain *Lactobacillus* species like *L. plantarum* and *L. brevis*, are known to produce GABA, an inhibitory neurotransmitter crucial for regulating neuronal excitability (56, 57). These bacteria, including *Streptococcus*, *Enterococcus*, and *Escherichia coli*, have been implicated in 5-HT production. 5-HT is primarily synthesized in the gut and plays a significant role in mood regulation (55, 58). Some bacteria, such as *Bacillus* species, are known to produce DA, which has the ability to reinforce reward-causing behaviors, affect attention, memory, and mood, but *Escherichia coli* may influence dopamine levels indirectly through their metabolic activities (55, 59). NE is a neurotransmitter involved in attention, arousal, and executive functions. It helps regulate the brain’s ability to focus and maintain attention, which are often impaired in ADHD (60). Several types of gut flora have been identified as capable of producing NE, such as *Bacillus mycoides, Bacillus subtilis, Proteus vulgaris, and Escherichia coli* (55, 59, 61). In addition, gut microbiota affects the nervous system partially through bacterial metabolites, especially short-chain fatty acids (SCFAs). SCFAs are the main metabolites produced in the colon by bacterial fermentation of dietary fibers and resistant starch (62), which impact neurodevelopment, immune signaling, and the integrity of the blood-brain barrier (BBB) (63). ADHD patients (both children and adults) have significantly lower SCFA levels compared to healthy controls, with medicated individuals showing the most pronounced reductions (64, 65). Lower SCFAs may contribute to pro-inflammatory states, as ADHD patients exhibit elevated immune markers (e.g., sICAM-1, sVCAM-1) alongside SCFA deficits (65). The mechanisms by which short-chain fatty acids affect ADHD include the following three areas: SCFAs influence dopamine, serotonin, and norepinephrine signaling—key pathways disrupted in ADHD (66, 67). SCFAs regulate gut barrier integrity and interact with vagal nerve signaling, indirectly influencing brain function (64, 66). SCFAs like butyrate modulate immune responses and reduce neuroinflammation, which is implicated in ADHD pathophysiology (64, 67).

The gut-brain axis signal transduction mechanism involves two primary barriers: the intestinal barrier and BBB. The intestinal barrier serves as the first line of defense between the human body and the intestinal lumen, and its function impacts the transport of substances between the body and the intestinal lumen, as well as the invasion of pathogens (68). Disturbance of gut microbiota increases intestinal permeability by disrupting mucosal surface biofilms, leading to lipopolysaccharide translocation and hyperactivation of the immune system. This results in elevated levels of pro-inflammatory cytokines (69). Excessive release of pro-inflammatory cytokines is detrimental to the CNS (70). SCFAs, including propionic, butyric, and acetic acids, are end products of bacterial fermentation and function as signaling molecules with anti-inflammatory properties. They also protect colonic epithelial cells (71). Numerous studies indicate that SCFAs maintain barrier integrity, protect the BBB from oxidative stress, and promote tight junction protein expression (72, 73).

Gut microbiota also modulates the permeability and integrity of the BBB. The BBB plays an important role in regulating the exchange between the cerebrospinal fluid and the circulatory system, helping to maintain the stability of the internal environment of the CNS (74). The gut microbiota influences the permeability of BBB by regulating the expression of tight junction proteins (75). Research on mice indicates a heightened permeability of the BBB in germ-free (GF) compared to normal mice (76, 77). Subsequent studies have demonstrated that treatment with sodium butyrate can effectively restore tight junction proteins and reduce BBB permeability in GF mice (78).

An increasing number of studies suggest a close relationship between alterations in gut microbiota diversity and composition and various central nervous system diseases, including ADHD, ASD, obsessive-compulsive disorder, Alzheimer’s disease, Parkinson’s disease, and multiple sclerosis (79, 80). Numerous studies have demonstrated that the composition of the gut microbiome in individuals with various psychiatric disorders differs from that of healthy individuals (81, 82). By meticulously regulating disordered gut microbiota, it is possible to witness a notable improvement in neurological functioning to a certain extent (83). In summary, gut microbiota is crucial in maintaining the balance between health and disease, particularly in relation to brain function and behavior.

## 4 Gut microbiota characteristics of children with ADHD

Recent studies have explored the relationship between childhood ADHD and the gut microbiome. Among these, two studies have linked the composition of the gut microbiome in infancy to the development of ADHD in children. In 2015, Pärtty et al. showed that babies with higher levels of *Bifidobacterium* in their gut microbiota had a lower risk of developing ADHD in the future (84). In 2023, Cassidy-Bushrow et al. published a birth cohort study showing that early life gut microbiome characteristics were linked to ADHD development by age 10. The gut microbiota of children with ADHD at 6 months of age was different, characterized by a lack of lactic acid bacteria like *Lactobacillales* and *Enterococcaceae* (85). These two prospective studies indicate that focusing on the microecological composition of the gut early in life is crucial for the development of ADHD. Concurrently, an increasing number of scientists are conducting cross-sectional studies to examine the changes in gut microbiota of children with ADHD.

In 2017, Aarts et al. analyzed the gut microbiota of ADHD patients and controls using 16S rRNA. They found a decrease in the abundance of *Firmicutes* and an increase of *Actinobacteria* and *Bifidobacterium* in ADHD patients (86). In 2018, Prehn-Kristensen et al, discovered a notable decrease in gut microbiota diversity in boys with ADHD, and the composition varied greatly between patients and controls (87). ADHD children exhibited elevated levels of *Bacteroidaceae*, *Neisseriaceae* and *Neisseria* sp*ec* have been identified as potential indicators of ADHD in adolescents. However, the study’s limitation lies in its inability to exclude the drug’s effects on the gut microbiota (87). Jiang et al. demonstrated a significant reduction in *Faecalibacterium* in the gut microbiota of children with ADHD, which was negatively associated with symptoms reported by parents (88). In 2019, Stevens et al. found a strong association between the relative ADHD symptom scores and abundance of *Bifidobacterium* (89). In 2020, Wang et al. reported elevated gut microbiota diversity in children with ADHD, along with decreased relative abundance of *Bacteroides coprocola* and increased relative abundance of *Bacteroides ovatus*, *Sutterella stercoricanis*, and *Bacteroides uniformis* (79). In 2020, Szopinska-Tokov et al. continued to expand the sample size based on the study by Aarts et al. and seven genera of gut microbiota were found to be correlated with ADHD symptom scores in patients with ADHD (86, 90).

In 2022, Wang et al. revealed that children with ADHD had higher levels of *Agathobacter, Anaistipes* and *Lachnospiraceae UCG-010*, lower levels of plasma TNF-α levels than healthy children, and a negative correlation between TNF-α levels and ADHD symptom scores and gut microbiota diversity (91). In the same year, Lee et al. discovered that *Agathobacter* in the gut microbiota was linked to withdrawal and depressive symptoms in ADHD children, while *Ruminococcus gnavus* was associated with rule-breaking behavior (92). In 2023, Bundgaard-Nielsen et al. found that the relative abundance of *Eggerthella* was higher in children with ADHD, in agreement with the results of Aarts (86). The relative abundance of *Colobacterium* spp、*Cholera* spp and *Hodgkinella* spp was low, which is consistent with the findings of Prehn-Kristensen (86, 87, 93).

All these studies noted differences in the gut microbiota composition in children with ADHD based on 16S rRNA, but their results may be limited by differences in population and dietary patterns. Metagenomic technology has advanced, enabling an increasing number of ADHD studies to utilize it, thereby improving the accuracy of results.

In 2020, Wan et al. found no significant differences in alpha diversity in gut microbiota of children with ADHD and healthy children, but significant differences in the composition of the microbiota. Compared to healthy children, ADHD had substantially reduced abundances of *Faecalibacterium* and *Veillonellaceae*, and markedly increased abundances of *Odoribacter* and *Enterococcus*. At species level, the abundance of R*uminocococockus gnavus*, *Faecalibacterium prausnitzii* and *Lachnospiraceae bacterium* in children with ADHD decreased significantly, while the abundance of *Paraprevotella xylaniphila, Odoribacter* sp*anchnicus, Veillonella parvula*, and *Bacteroides caccae* increased significantly (94). The reduced abundance of *Faecalibacterium* observed in this study is consistent with the findings of Jiang et al. (88).

In 2022, Li et al. through metagenomic technology from 207 fecal samples that the children’s gut microbiome with ADHD was more abundant in *Prevotella* (*amnii, buccae* and *copri*) and *Bifidobacterium* (*breve* and *bifidum*) (95). This result is consistent with the findings of Aarts (66). In addition, the presence of distinguishable gut microbiota patterns in ADHD subgroups predicts that more accurate gut microbiota information can be obtained through subgroup analysis. It was further shown that *Bacteroides ovatus* supplementation improved spatial working memory deficit and reversed EEG rhythm in ADHD rats (95). In 2023, Stiernborg et al. investigated the impact of neuroleptics on the ADHD gut microbiome and found that children with drug-treated ADHD had significantly different β-diversity, lower functional gut microbiota, and lower abundance of the strain *Bacteroides stercoris CL09T03C01* and bacterial genes encoding an enzyme in vitamin B12 synthesis, as compared to the control group (96). In 2023, Wang et al. discovered variations in β-diversity between children with ADHD and healthy controls by high-throughput next-generation sequencing, with significantly increased abundance of *Ascomycetes* and *Candida* in children with ADHD (97). In 2024, Wang et al. found that the gut microbiota of children with ADHD was characterized by enrichment of *Anaerostipes_hadrus*, *Lachnospira*, and *Phascolarcto - bacterum_ faecium*, which might help identify potential biomarkers of ADHD (98). Changes in the gut microbiome of children with ADHD are presented in **Supplementary Table 1**.

**Figure 1:**
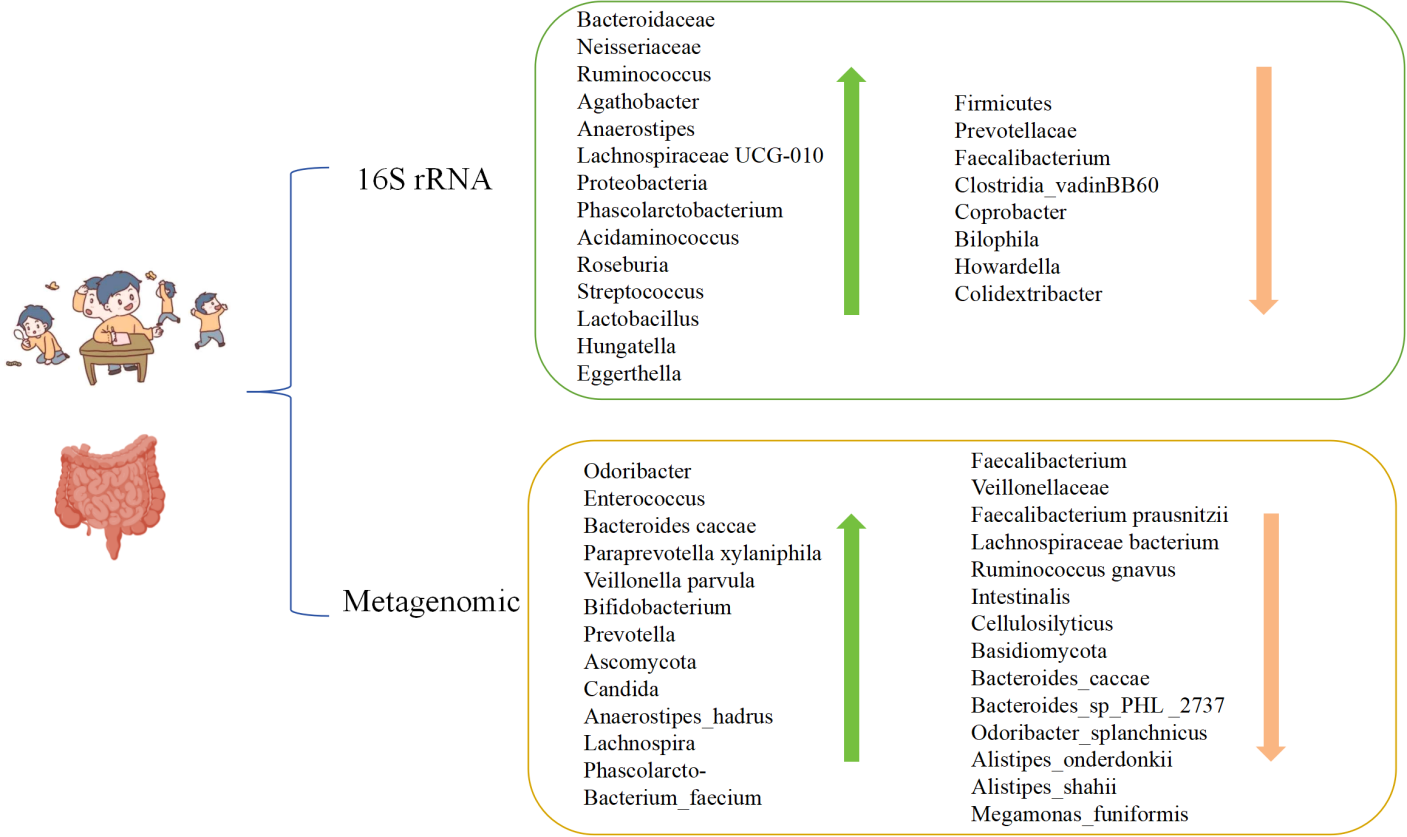
The changes of gut microbiota in children with ADHD using different testing methods.

Although the above studies of the gut microbiome in children with ADHD vary due to differences in populations inclusion, sample sizes, and testing methods, leading to diverse findings and even contradictory conclusions, upon more detailed analysis, there has been some evidence of altered gut microbiota in children with ADHD using different testing methods (**Figure 1**). Therefore, it is important to explore the possible mechanisms by which gut microecology affects ADHD.

## 5 Possible mechanism of gut microbiota imbalance affecting ADHD

The gut microbiota comprises trillions of microorganisms residing in the human intestine, including bacteria, archaea, fungi, viruses, protozoa, and other organisms, that exist in the human intestinal environment (67, 99). The gut microecology provides the host with various functions, such as the production of vitamins, absorption of calcium ions, resistance against pathogens, and enhancement of immune function (100, 101). Growing evidence suggests that gut microecology affects brain development and function (102–104). Gut microecological disorders may directly or indirectly affect neural function through neural, metabolic, immune pathways and vice versa (105).

### 5.1 Nerve pathway

Control of the intestine is by the autonomic nervous system (ANS) and ENS. The vagus nerve, the 10th cranial nerve, is a major component of the ANS and plays a critical role in emotion regulation (106), and stress regulation by affecting the hypothalamic-pituitary-adrenal (HPA) axis (49). Mouse models had demonstrated that activation of gastrointestinal vagal afferents influenced rewarding behavior (67), while vagotomy attenuated aggressive behavior in ADHD rats (107). Gut microbiota can modulate host emotional and behavioral responses via vagal afferents. Pathogenic infections such as *Campylobacter jejuni* and *Citrobacter aminopropionate* induced anxiety-like behaviors in animal models (108). Long-term intake of *Lactobacillus rhamnosus GG* (LGG) can regulate the mRNA level of GABA receptors via the vagus nerve, thereby alleviating anxiety symptoms in mice (109).

Recent studies have also reported potential mechanisms for the interaction between the gut microbiota and ENS. ENS is a complex network of neurons and glial cells that communicate with CNS through afferent neurons that carry sensory information along the spinal cord and vagus nerve pathways. The ENS also controls secretion and motility in the gastrointestinal tract, which contributes to the maintenance of the intestinal barrier integrity (110). ENS dysfunction is associated not only with a variety of gastrointestinal problems, but also with an increasing number of CNS illnesses, such as ASD (111). It has been shown that ENS changes occur in the early stages of some neurological diseases, even prior to overt CNS involvement (112). ENS maturation and function are influenced by gut microecology, which may involve activation of pattern recognition receptors, including Toll-like receptors and 5-HT expression (113, 114). Antibiotic-mediated gut microbiota disturbance leads to reduced intestinal glial cell number, ENS structural and functional changes in mice (115). A different study likewise found that GF mice’s myenteric plexus’s AH neurons were less excitable, leading to decreased gastrointestinal motility, indicating gut microbiota is crucial for ENS development (116). Animal experiments further showed that restoring gut microbiota could effectively improve the intestinal function of antibiotic-induced mice and increase the number of intestinal glial cells and neurons, while supplementation of SCFAs could restore neuronal loss caused by antibiotics (117). The irregular excitability of the ENS caused by gut microbiota imbalance was also significantly reduced after supplementation with *Bifidobacterium longum* (118). The above studies indicate that gut microecology may further alter host mood and behavior patterns by affecting the ANS and/or ENS, which is of great significance for understanding gut-brain axis interaction, especially ADHD pathogenesis.

### 5.2 Neuroendocrine pathway

ADHD-related brain networks are innervated by neurotransmitters such as DA, NA, GABA and 5-HT et al. Impaired reward sensitivity, issues with attention and working memory, and poor behavioral regulation (hyperactivity and impulsivity) can all result from disorders involving these neurotransmitters (60, 119–122). The synthesis and control of multiple neurotransmitters are concurrently linked to the gut flora. The gut microbiota communicates with CNS by producing neurotransmitter precursors/neurotransmitters or metabolites. Among them, DA, 5-HT, GABA and SCFAs are crucial in the development of disorders affecting the neurological system (16, 95). DA receptors, particularly D1 and D2, are involved in signaling reward circuits, learning, and memory. A higher density of the DA transporter in ADHD patients can lead to altered dopamine levels in the synaptic cleft, affecting cognitive processes (123). 5-HT influences mood and impulse control. Increasing serotonin levels can help manage ADHD symptoms related to impulsivity and hyperactivity (124, 125). GABA is the primary inhibitory neurotransmitter, helping to reduce neuronal excitability. Lower GABA levels have been linked to increased impulsivity in ADHD (126). SCFAs, produced by gut microbiota, play a role in the gut-brain axis. They influence glucose metabolism and immune regulation, potentially impacting brain function (64, 127).

Catecholamines, including DA and NA, are key neurotransmitters that influence mood, behavior, and a host of other processes in the central and peripheral nervous systems (128–130). The gut microbiome has the capacity to create DA, NA, and their precursors (131). Asano et al. found that the intestinal lumen of GF mice had lower levels of free DA and NA than those of specific pathogen-free (SPF) animals. The levels of DA and NA in the intestinal lumen significantly increased in GF mice after receiving fecal transplantation from SPF mice, suggesting that gut microbiota is involved in the generation of intestinal catecholamines (132). In addition, *Prevotella* and *Bacteroides* in the intestine can indirectly affect DA bioavailability by directly affecting DA transporters or changing the level of intestinal peptide hormones (133). Oral administration of *Lactobacillus plantarum PS128* can improve DA-related neurological diseases (134). Aarts et al. discovered a negative correlation between cyclohexadienyl dehydratase and reward anticipatory responses, as well as a significant increase in the relative abundance of the *Bifidobacterium genus* in fecal samples from ADHD patients. This genus is involved in the synthesis of the dopaminergic precursor, phenylalanine (86). An increasing amount of evidence indicates that the gut bacteria may be involved in the production and breakdown of NA. It has shown that *E. coli K-12, Bacillus* spp and *Saccharomyces spp* can produce NA (55). In the brain, NA inhibits the transcription of inflammatory genes and increases the level of brain-derived neurotrophic factor produced by microglia and astrocytes, thus further promoting neuronal survival and neuroprotection (135).

GABA is critical to the pathophysiology of ADHD. Children with ADHD have been shown to have lower GABA levels, and GABA imbalances are associated with reduced ability to focus on high-difficulty tasks (136, 137). *Bifidobacterium*, *Lactobacillus* and *Escherichia coli* have been documented to generate GABA (138, 139). By raising GABA levels, early *Lactobacillus* intervention can lower the incidence of ADHD in later life (84, 140).

The neurotransmitter 5-HT is crucial for neuronal transmission and is implicated in the etiology of ADHD. Children with ADHD exhibit lower 5-HT levels compared to healthy individuals, and the primary symptoms of ADHD, such as impulsivity, hyperactivity, and inattention, are linked to these low levels of 5-HT (141). Although there is no evidence that 5-HT levels in the gut are directly related to ADHD brain function, approximately 90% of 5-HT in the body is produced by Enterochromaffin cells, and the gut microbiota can alter the utilization of the availability of tryptophan, a 5-HT precursor (142, 143). Tryptophan can cross the BBB and affect 5-HT synthesis in the central nervous system (144). Clinical studies have demonstrated that ADHD patients have lower 5-HT concentrations, consistent with observations in GF mice, and tryptophan supplementation can alleviate ADHD-related symptoms (145).

SCFAs are gut microbiota products that degrade cellulose and dietary fibers (146). High-dose propionic acid administration during pregnancy alters social behavior in rat pups during the neonatal period and adolescence (147). Studies have found lower plasma and fecal SCFA levels in ADHD patients compared to healthy controls. Propionic acid levels have been negatively correlated with ADHD symptom severity, suggesting its potential as a biomarker (64, 127). Butyrate possesses anti-inflammatory properties, inhibiting pro-inflammatory cytokines synthesis such as IL-6 and IL-12, and inducing anti-inflammatory cytokine IL-10 release (148). Another study revealed SCFAs improved reduced microglia activity in GF mice (149). Psychostimulant medications, commonly used to treat ADHD, may alter gut microbiota composition and SCFA levels, potentially affecting the gut-brain axis and treatment outcomes (64).

The HPA axis is the key to neuroendocrine transmission and stress response systems, and one study found abnormal HPA axis function in severely hyperactive ADHD children (150). Another study finds delayed HPA axis response to stress linked to ADHD impulsive behavior (131). The HPA axis’s growth and regulation can be influenced by gut bacteria (49). The HPA axis of GF mice is over-responsive to restraint stress, and early administration of *Bifidobacterium* is effective in reversing the excessive HPA stress response in GF mice (151).

The above studies suggest that gut microbiota impacts the nervous system by influencing neurotransmitters or SCFAs, and that this pathway may also be an important way in which disordered gut microbiota impacts ADHD.

### 5.3 Immune pathway

The gut microbiota plays a critical role in regulating the host immune system and mitigating inflammation and immune-mediated illnesses (152–154). Dysbiosis, or alterations in the gut microbiota, can disrupt tight junction protein expression and trigger neuroinflammatory responses (78, 155), impairing both intestinal barrier and blood-brain barrier (BBB) function (156).

Disruption of the BBB is a proposed mechanism for neurological disorders including ADHD (157, 158). Microglia are critical for regulating BBB integrity and function. The brain’s immune system is primarily composed of microglia. Microglia participate in neuroinflammatory processes and brain homeostasis regulation. Their development and maturation depend highly on gut microbiota (42, 159). It has been shown that reduced gut microbiota diversity can cause microglia dysfunction linked to neurodegenerative diseases or behavioral disorders, via neuroinflammation (42).

**Figure 2:**
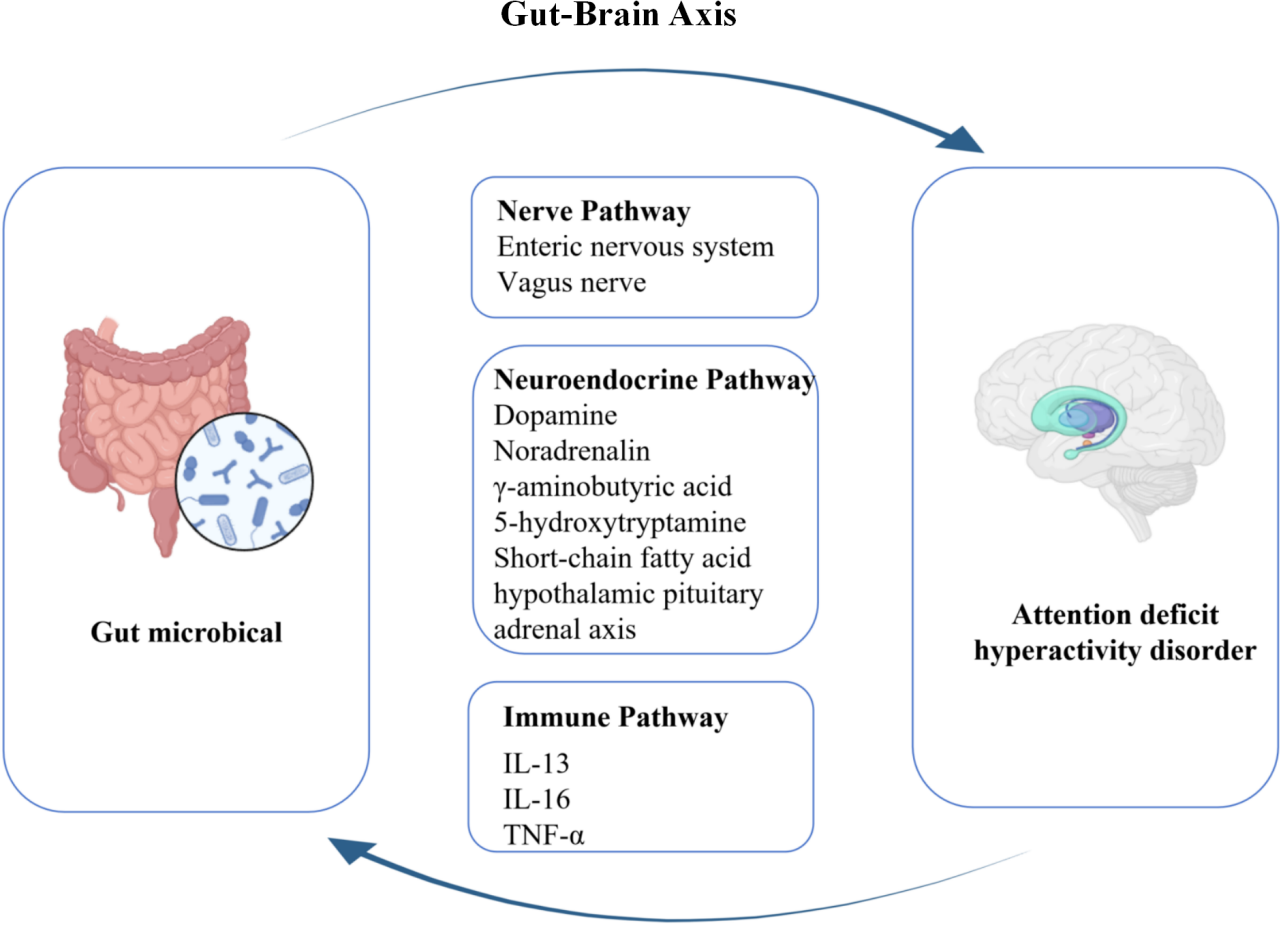
The possible mechanisms of gut microbiota in influencing ADHD via the gut-brain axis. IL, Interleukin; TNF-α, Tumor Necrosis Factor-α.

There is evidence that people with ADHD have a significantly increased risk of atopic dermatitis, allergic rhinitis, eczema, and autoimmune diseases (32). Individuals with atopic disorders have a significantly increased risk of developing ADHD in the future, ranging from 30-50%, compared to non-atopic children (32). A meta-analysis finds mild inflammation in peripheral cytokine levels in patients with ADHD (160). ADHD children had higher levels of circulating pro-inflammatory cytokines, including TNF-α, IL-16, and IL-13 (161, 162). Another study found that behavior in ADHD patients was associated with inflammatory markers, with IL-13 levels linked to inattention and IL-16 levels linked to hyperactivity (161). The possible mechanisms by which gut microbiota affects ADHD through various pathways are shown in **Figure 2**.

## 6 Microecological agent in ADHD

According to the disruption of the gut microbiota in ADHD children, the gut microbiota affects neurodevelopmental disorders, especially ADHD, through the gut-brain axis. Thus, targeting the gut microbiota has become a cutting-edge treatment strategy for ADHD. Currently, the common types of microecological agents are probiotics, prebiotics and synbiotics.

### 6.1 Probiotics

Probiotics are microorganisms that benefit the host and improve or restore gut microbiota. Currently, the clinical applications of probiotics mainly include inflammatory bowel disease, lactose intolerance, allergies and neurological diseases (163). Research indicates probiotics may positively impact Alzheimer’s disease and Parkinson’s disease by modulating the gut-brain axis, neurotransmitter production, and reducing neuroinflammation, as well as alleviating anxiety, depression and cognitive disorders. They may also have clinical benefits for children with ADHD (164).

In 2015, Pärtty et al. published the first study on probiotics for preventing ADHD, which showed that early LGG treatment decreased the incidence of ASD and ADHD by the age of 13 (84). In 2017, Bazinet et al. conducted a double-blind, randomized, placebo-controlled research to investigate the impact of probiotic supplementation on children’s anxiety, ADHD, and memory (165). Probiotics contained *Bifidobacterium longum R0175* and *Lactobacillus helveticus R0052*, the treatment course was 13 weeks. A total of 48 children with ADHD (6–15 years old) participated in the study, which showed that probiotic supplementation did not significantly improve symptoms in children with ADHD as a whole. However, after excluding children treated with stimulants, those who were not treated with medication showed a reduction in hyperactivity and inattentiveness, and an improvement in memory functioning (165). This suggests that the effects of probiotics on ADHD may be influenced by other medications.

In 2020, Kumperščak et al. published a randomized, double-blind, placebo-controlled trial enrolling 32 children and adolescents with ADHD (aged 4–17 years), examining changes in ADHD symptoms, the Child Self-Report of the Pediatric Quality of Life Inventory™, and serum cytokine levels in the two groups before LGG treatment and after 3 months of treatment (166). The study showed a significant improvement in The Quality of Life scores after 3 months of LGG supplementation in children with ADHD, whereas there was no substantial change in the group receiving a placebo. Nonetheless, there were no variations between the two groups’ hardcore symptoms, mood, or behavior. TNF-α and IL-12 p70, two pro-inflammatory serum cytokines, were only lower in the probiotic group (166). The findings imply that LGG supplementation might be advantageous, but more thorough investigation with a longer observation period and more participants is necessary due to the variations in inflammatory cytokine levels.

In 2021, Ahmadvand et al. included 34 children with ADHD (8–12 years) in a randomized, double-blind, placebo-controlled trial (167). All children were randomly assigned to receive either probiotic supplement (n=17) or placebo (n=17) for 8 weeks on top of Ritalin, a stimulant for the treatment of ADHD. The probiotics included *Bifidobacterium bifidum, Lactobacillus fermentum, Lactobacillus acidophilus*, and *Lactobacillusreuteri.* At the start of the trial and after 8 weeks of treatment, clinical symptoms were documented using the Hamilton Anxiety Rating Scale (HAM-A), Children’s Depression Inventory (CDI), and rating scale of ADHD (ADHD-RS). In addition, blood samples were taken at the start and eight weeks to assess metabolic information. The results showed that the addition of probiotics positively affected the levels of ADHD-RS, HAM-A, C-reactive protein in serum and total antioxidant capacity, while there was no effect on CDI and other metabolic profiles (167). This study was the first clinical study to explore the effect of probiotic supplementation on children with ADHD who were undergoing Ritalin treatment. Unfortunately, the research was not able to evaluate changes in gut microbiota and its gene expression related to inflammation and oxidative stress to clarify the pathogenesis.

In 2022, Wang et al. used *Bifidobacterium bifidum* (Bf-688) for the first time as ADHD supplementation (168). 30 ADHD children, ages 4 to 16, completed this single-arm, open-label trial, and all subjects received Bf-688 daily for 8 weeks. Fecal samples were collected for gut microbiota testing at baseline, the 4th and 8th weeks. At the 4th and 8th weeks of Bf-688 supplementation, the children with ADHD showed improvements in their symptoms of inattention and hyperactivity/impulsivity, as well as a rise in their weight and BMI. Gut microbiota testing revealed a significant decrease in the ratio of *Firmicutes* to *Bacteroidetes* (F/B) from baseline to week 8. Throughout the course of the 8-week treatment, *Firmicutes* significantly declined while *Proteobacteria* greatly rose. *Bacteroidota* considerably declined and *Shigella* greatly rose after Bf-688 was stopped for four weeks. The authors concluded that Bf-688 significantly improved clinical symptoms in children with ADHD by affecting the gut microbiota’s makeup. However, the research was a single-arm trial, ADHD symptom scoring was relatively subjective, and the sample size was insufficient for performing a subgroup analysis, so the results need further verification.

In 2022, Ghanaatgar et al. used compound probiotics as a supplement to Ritalin for the treatment of ADHD (169). 38 children between the ages of 6 and 12 finished the double-blind, randomized, placebo-controlled study. The probiotic capsules used in the study contained 14 strains and were administered for 8 weeks. The Conners Parent Rating Scale–short version (CPRS–RS) and the Clinical Global Impression–Severity (CGI–S) scale were collected from all subjects before the intervention, at the conclusion of four weeks and eight weeks. When compared to the placebo group, the compound probiotic group’s CPRS-RS and CGI-S outcomes were noticeably better. This study clearly showed that compound probiotics, as a supplement to Ritalin, could provide clinical benefits to children with ADHD. However, the study lacked gut microbiota testing results and was not able to follow up long-term after treatment. As a result, care should be taken when extrapolating the findings to the whole ADHD population. Detailed clinical studies on probiotics for the treatment of ADHD are shown in **Table 1**.

**Table 1:** Clinical study on probiotics treatment for children with ADHD.

Research type	Study (year)	Object	Probiotic type	Probiotic dosage	Duration	Result
Randomized, Double-Blind, Placebo-Controlled	Pärtty (70) (2015)	Cases (n=40) Control (n=30)	*Lactobacillus rhamnosus* GG (ATCC 53103)	not provided	first 6 months of life	Reducing the risk of ADHD and ASD at 13 years of age
Randomized, Double-Blind, Placebo-Controlled	Bazinet (147) (2017)	Cases (n=25) Control (n=23)	*Lactobacillus helveticus R0052 Bifidobacterium longum* R0175	not provided	13 weeks	Improvements in symptoms and memory function in children with ADHD who were not treated with stimulant medications
Randomized, Double-Blind, Placebo-Controlled	Kumperščak (148) (2020)	Cases (n=18) Control (n=14)	*Lactobacillus rhamnosus* GG (ATCC53103)	at least 10^10^CFU	3 months	Improved health-related quality of life in children with ADHD
Randomized, Double-Blind, Placebo-Controlled	Ahmadvand (149) (2021)	Cases (n=17) Control (n=17)	*Lactobacillusreuteri* *Lactobacillus acidophilus* *Lactobacillus fermentum* *Bifidobacterium bifidum*	8×10^9^CFU	8 weeks	ADHD−RS, HAM−A, and hs−CRP decreased; TAC level increased
Single-arm trial	Wang (150) (2022)	Cases (n=30)	*Bifidobacterium bifidum* (Bf-688)	5×10^9^CFU	8 weeks	Improvement in inattention symptoms and hyperactivity/impulsivity symptoms, as well as weight gain
Randomized, Double-Blind, Placebo-Controlled	Ghanaatgar (151) (2022)	Cases (n=21) Control (n=17)	*Bacillus subtilis* PXN 21, *Bifidobacterium bifidum* PXN 23 *Bifidobacterium breve* PXN 25 *Bifidobacterium infantis* PXN 27 *Bifidobacterium longum* PXN 30 *Lactobacillus acidophilus* PXN 35 *Lactob. delbrueckii ssp* *Bulgaricus* PXN 39 *Lactob. casei* PXN 37 *Lactob. plantarum* PXN 47 *Lactob. rhamnosus* PXN 54 *Lactob. helveticus* PXN 45 *Lactob. salivarius* PXN 57 *Lactococcus lactis ssp* *lactis* PXN 63 *Streptococcus hermophiles* PXN 66	2×10^9^CFU	8 weeks	CPRS–RS and CGI–S were improved

CFU, colony-forming-unit; ADHD, attention deficit hyperactivity disorder; ASD, autism spectrum disorders; ADHD−RS, rating scale of ADHD; HAM−A, hamilton anxiety rating scale; Hs-CRP, high-sensitivity c-reactive protein; TAC, total antioxidant capacity; CPRS–RS, conners parent rating scale–short version; CGI–S, clinical global impression–severity.

In 2024, Yin et al. found in an animal research that *Bifidobacterium animalis subsp. lac*tis *A6* (BAA6) could ameliorate hyperactivity symptoms and spatial memory deficits in ADHD rats, increase the number of neurons in the hippocampus, elevate neurotransmitters levels such as DA, NA and ACh in brain tissue, improve brain-derived neurotrophic factor and reduce glutamate (170). *Lactobacillus*, *Romboutsia*, *Blautia*, and *Turicibacter* were significantly enriched in the intestinal tract of ADHD rats after BAA6 intervention (170). This study shows that BAA6 has great potential as a dietary supplement that may improve memory dysfunction associated with ADHD through the gut-brain axis.

### 6.2 Prebiotics/synbiotics

Non-digestible components of food called prebiotics specifically increase the action of probiotics that are already in the intestines. When combined, probiotics and prebiotics form synbiotics. While there is a lack of clinical or basic research on prebiotics as a treatment for ADHD. In 2020, Skott et al. published a randomized, double-blind, controlled trial to evaluate the impact of Synbiotic 2000 on symptoms of autism, ADHD, and functional impairment (171). For 9 weeks, 182 children and adults received either Synbiotic 2000 or a placebo daily. The outcomes showed that both Synbiotic 2000 and the placebo reduced ADHD symptoms. There were no significant differences in ADHD functioning or autistic symptoms between the two groups. Although no clear specific effects of Synbiotic 2000 were observed, an examination of patients with elevated levels of soluble vascular cell adhesion molecule-1, suggested that Synbiotic 2000 might improve autism symptoms in children and regulate mood in adults with ADHD.

## 7 Fecal microbiota transplantation

FMT is the process of transferring healthy donor feces into a patient (receiver) in order to balance the gut microbiota, mend the intestinal mucosal barrier, manage inflammation, and adjust the immune system. Studies have demonstrated that transplantation of fecal microbiota from patients with different mental illnesses into GF mice results in abnormal behavior (172–174). Another study found that transplanting the fecal microbiota of ADHD patients into mice not only altered the composition of gut microbiota, but also their brain structure, functions and behavioral (175).

There are currently no studies investigating FMT for children with ADHD. The only case report on FMT for adult ADHD was published by Hooi et al. in 2022 (176). A 22-year-old woman who experienced multiple episodes of severe diarrhea due to a recurrent *Clostridioides difficile* infection. Following TMT treatment, comorbid ADHD and anxiety symptoms were alleviated, with the CGI–S dropping from 5 points before treatment to 2 points. Fecal samples were subjected to metagenomic sequencing both before and after FMT, and the results revealed that a total of 106 bacteria were elevated or colonized and 59 bacteria were reduced or eradicated after treatment. Three prominent engrafted species that have been negatively associated with ADHD in the past include *Faecalibacterium prausnitzii*, *Lactobacillus ruminis*, and *Lactobacillus rogosae*. Important species linked to the pathophysiology of ADHD, including *Bacteroides ovatus*, *Bacteroides uniformis*, *Enterococcus avium*, *Enterococcus gallinarum*, *Fusobacterium ulcerans*, *Bifidobacterium adolescentis*, *Bifidobacterium animalis*, *Bifidobacterium breve* and *Bifidobacterium longum*, were all found to be diminished after FMT. Although the patient in this report was a 22-year-old adult female, this case provides hope for the future of FMT treatment for children with ADHD.

The studies mentioned above show that microecological agents have demonstrated positive therapeutic effects in the prevention and treatment of ADHD: supplementing with LGG in early life can significantly reduce the risk of ADHD、compound probiotics as a Ritalin supplement can dramatically improve the symptoms in children with ADHD、the advantages of FMT for adult ADHD may open new directions for the treatment of children with ADHD in the future.

## 8 Conclusion

In summary, there is an alteration in the gut microbiome of children with ADHD, and the gut microbiota can affect ADHD through neural, endocrine, and immune pathways. Therefore, targeting the gut microbiota, with microecological agents or FMT, especially in combination with central nervous system stimulants, may provide additional benefits for children with ADHD. Future, large-scale, randomized, controlled clinical trials of microecological agent or FMT are needed to explore their effectiveness and safety for children with ADHD.
