# Supplementary material for: A narrative review of research advances in gut microbiota and microecological agents in children with attention deficit hyperactivity disorder (ADHD)
Source: Front Psychiatry. 2025 May 23;16:1588135. doi: 10.3389/fpsyt.2025.1588135 (PMC12141249; doi:10.3389/fpsyt.2025.1588135)
Supplement: Supplementary file 1 [file Table1.docx]

Supplement Table 1: Changes of gut microbiota in children with ADHD.

| Year | Researcher | Object | | Method | Upgrade | Downgrade |
| --- | --- | --- | --- | --- | --- | --- |
| 2017 | Aarts [72] | cases 19  control 77  (17.0- 31.4 year) | | 16S rRNA | *Actinobacteria*  *Bifidobacterium* | *Firmicutes* |
| 2018 | Prehn-Kristensen [73] | cases 14  control 17  （11.9-13.1 years） | | 16S rRNA | *Bacteroidaceae*  *Neisseriaceae*  *Neisseria spec* | *Prevotellacae* |
| 2018 | Jiang [74] | cases 51  control 32  （ 6- 10 years） | | 16S rRNA |  | *Faecalibacterium* |
| 2019 | Stevens [75] | cases 7  control 17  （7-12 years） | | 16S rRNA |  | *Actinobacteria*  *Bifidobacterium* |
| 2020 | Wang [65] | cases 30  control 30  （8.4-9.3 years） | | 16S rRNA | *Bacteroides uniformis*  *Bacteroides ovatus*  *Sutterella stercoricanis* | *Bacteroides coprocola* |
| 2020 | Szopinska-Tokov [76] | cases 42  control 50  （13-29 years） | 16S rRNA | | *Intestinibacter* | *Prevotella_9*  *Coprococcus_2* |
| 2022 | Wang [77] | cases 41  control 39  （6-16 years） | 16S rRNA | | *Agathobacter*  *Anaerostipes*  *Lachnospiraceae UCG-010* |  |
| 2022 | Lee [78] | cases 54  control 22  （6-18 years） | 16S rRNA | | *Proteobacteria*  *Agathobacter*  *Phascolarctobacterium*  *Prevotella_2*  *Acidaminococcus*  *Roseburia*  *Ruminococcus* |  |
| 2023 | Bundgaard-Nielsen [72] | cases 55  control 04  （5-17 years） | 16S rRNA | | *Streptococcus*  *Lactobacillus*  *Hungatella*  *Eggerthella*  *Ruminococcus* | *Clostridia_vadinBB60*  *Coprobacter*  *Bilophila*  *Howardella*  *Colidextribacter* |
| 2020 | Wan [80] | cases 17  control 17  （6-12 years） | Metagenomic | | *Odoribacter*  *Enterococcus*  *Bacteroides caccae*  *Odoribacter splanchnicus*  *Paraprevotella xylaniphila*  *Veillonella parvula* | *Faecalibacterium*  *Veillonellaceae*  *Faecalibacterium prausnitzii*  *Lachnospiraceae bacterium*  *Ruminococcus gnavus* |
| 2022 | Li [81] | cases 98  （7-11 years）  control 109  （7.1-10.7 years） |  | | *Bifidobacterium (breve and bifidum) Prevotella (amnii, buccae and copri)* | *Bacteroides (ovatus, fragilis)*  *taiotaomicron*  *Intestinalis*  *Cellulosilyticus*  *Salyersiae*  *Fluxus*  *Nordii* |
| 2023 | Stiernborg [83] | adult cases 84  adult control 52  children cases 33  children control 29  （ 5-55 years） | Metagenomic | |  | *Bacteroides stercoris CL09T03C01* |
| 2023 | Wang [83] | cases 35  control 35  （7.4-13.0 years） | High-throughput next-generation sequencing (NGS) | | *Ascomycota*  *Candida* | *Basidiomycota* |
| 024 | Wang [84] | cases 47  control 60  （6-16 years） | Metagenomics | | *Anaerostipes_hadrus*  *Lachnospira*  *Phascolarcto-bacterium_faecium* | *Bacteroides_caccae*  *Bacteroides_sp_PHL _2737*  *Odoribacter_splanchnicus*  *Alistipes_onderdonkii*  *Alistipes_shahii*  *Megamonas_funiformis* |
